# A Remitting Seronegative Symmetrical Synovitis With Pitting Edema (RS3PE) Syndrome Patient With High-Grade Serous Ovarian Cancer: A Possible Pathogenesis of Tumor-Derived Vascular Endothelial Growth Factor

**DOI:** 10.7759/cureus.48597

**Published:** 2023-11-10

**Authors:** Misa Itamura, Hikaru Kawahara, Naoki Sasaki, Natsuko Saito-Sasaki, Yu Sawada

**Affiliations:** 1 Dermatology, University of Occupational and Environmental Health, Kitakyushu, JPN

**Keywords:** literature review, case report, vegf, ovarian cancer, rs3pe syndrome

## Abstract

A 65-year-old female was previously diagnosed with remitting seronegative symmetrical synovitis with pitting edema (RS3PE) syndrome by internal doctors in our hospital nine years ago. Computed tomography revealed the presence of multiple disseminated peritoneal nodules with a large tumor mass. Histological analysis of the tumor and peritoneal nodules confirmed the diagnosis of high-grade serous ovarian cancer. The serum vascular endothelial growth factor (VEGF) level was highly elevated (1,223.9 pg/mL) (normal range: <38.3 pg/mL). One month after the first administration of docetaxel and cyclophosphamide chemotherapy, her peripheral edema decreased with a parallel reduction of serum VEGF (675.2 pg/mL). These findings suggest the correlation of VEGF with both RS3PE and ovarian cancer in this case.

## Introduction

Remitting seronegative symmetrical synovitis with pitting edema (RS3PE) condition is distinguished by bilateral synovitis and significant edema of the hands and feet [1]. Vascular endothelial growth factor (VEGF) is a potential humoral factor involved in tumor angiogenesis that has also been associated with RS3PE syndrome [2]. Herein, we present a case of RS3PE syndrome with ovarian cancer, possibly the source of the highly produced VEGF.

## Case presentation

**Figure 1:**
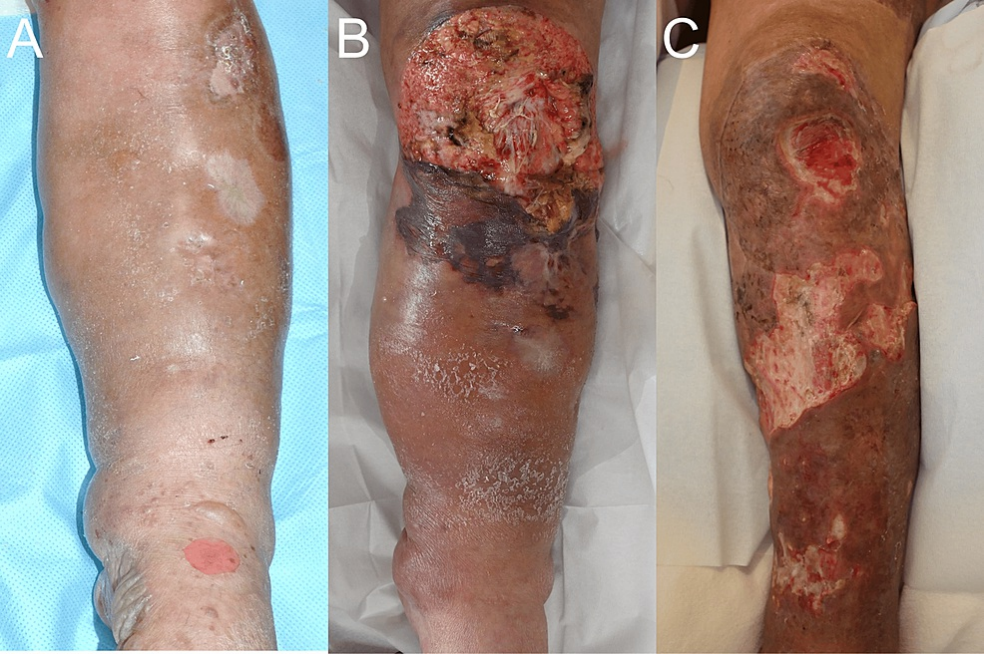
Clinical manifestations before (A) and after (B) the injury and of the resolution of peripheral edema (C)

**Figure 2:**
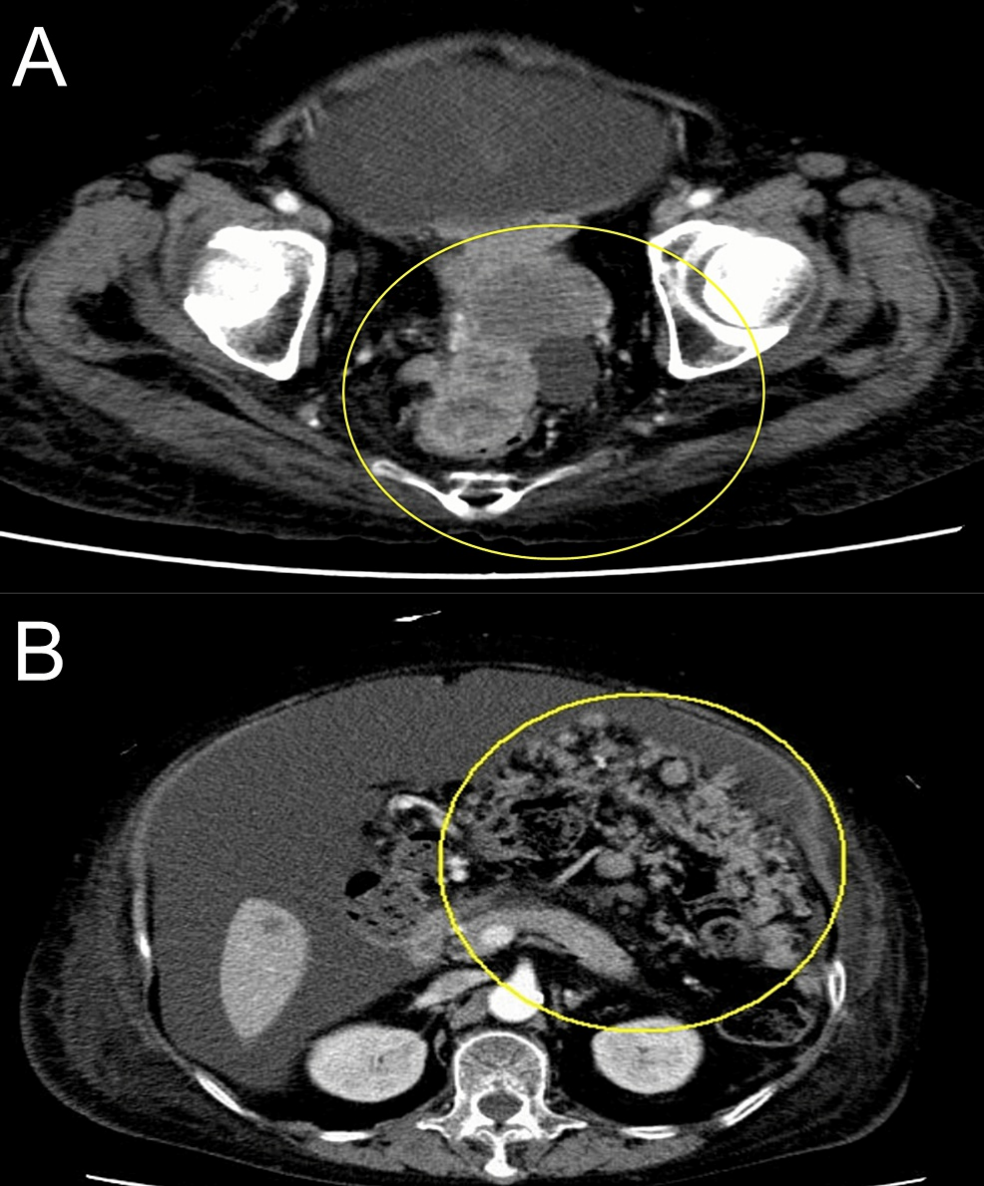
Computed tomography imaging of disseminated peritoneal nodules (A) and the large tumor (B)

**Figure 3:**
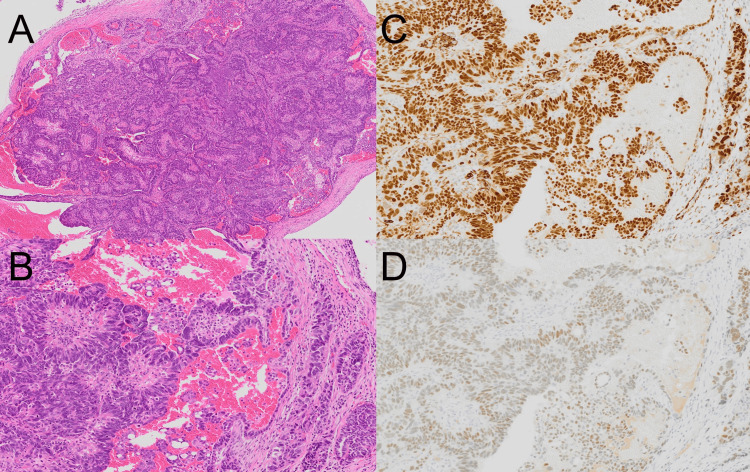
Histological examination (H&E staining ×20 (A) and ×100 (B)) and immunostaining (WT-1 (C) and estrogen receptor (D)) H&E: hematoxylin and eosin, WT-1: Wilms’ tumor 1

A 65-year-old female was previously diagnosed with RS3PE syndrome by internal doctors in our hospital nine years ago. Additionally, she was also treated with oral corticosteroid for bullous pemphigoid (Figure 1A). Until this moment, no malignancy had been identified by various examinations. She accidentally injured a large skin area of her left lower leg (Figure 1B), and a computed tomography screening test revealed the presence of multiple disseminated peritoneal nodules with a large tumor mass (Figure 2A, 2B). Histological analysis of the tumor and peritoneal nodules confirmed the diagnosis of high-grade serous ovarian cancer (Figure 3A-3D). At the moment, the serum vascular endothelial growth factor (VEGF) level was highly elevated (1,223.9 pg/mL) (normal range: <38.3 pg/mL). One month after the first administration of docetaxel and cyclophosphamide chemotherapy, her peripheral edema decreased with a parallel reduction of serum VEGF (675.2 pg/mL) (Figure 1C). These findings suggest the correlation of VEGF with both RS3PE and ovarian cancer in this patient.

## Discussion

RS3PE syndrome is a rare syndrome that causes peripheral edema; it was first described by McCarty in 1985 [1]. Although the entire etiology of RS3PE syndrome is still largely unknown, RS3PE syndrome patients showed a high serum VEGF level, which is the specific finding of RS3PE syndrome [2]. VEGF is a potent angiogenic vasoactive molecule that increases permeability to accelerate peripheral edema. Interestingly, the coexistence or precedence of malignant states has been identified in RS3PE syndrome. A recently updated study identified that 20% of cases present malignancies [3]. As far as we know, this is the first report indicating the correlation of VEGF with both RS3PE and ovarian cancer. Consistently, three cases of ovarian cancer with RS3PE syndrome have been reported, including our case (Table 1) [4,5]. Because ovarian cancer is difficult to identify during the early phase, the presence of ovarian cancer might need to be excluded in RS3PE syndrome cases by using more specific examinations such as transvaginal ultrasound tomography even if the computed tomography examination showed a negative result to identify malignancies.

**Table 1:** Literature review of RS3PE syndrome and ovarian cancer RS3PE: remitting seronegative symmetrical synovitis with pitting edema

First author	Age	Type of malignancy	Time to malignancy detection following RS3PE syndrome onset	Outcome of RS3PE syndrome
Vinci et al. [4]	69	Serous cystadenocarcinoma	8 months	No recurrence of edema
Kawano et al. [5]	46	Clear cell carcinoma	2 years	No recurrence of edema
Our case	65	High-grade serous carcinoma	9 years	Decreased peripheral edema after chemotherapy

## Conclusions

Taken together, we experienced a case of RS3PE syndrome with high-grade serous ovarian carcinoma. Following the diagnosis of RS3PE syndrome, a careful search for malignant tumors is essential in addition to the treatment of RS3PE syndrome.
